# Shoulder-Level Asymmetry Pre- and Post-Posterior Spinal Fusion in Adolescent Patients with Idiopathic Scoliosis

**DOI:** 10.3390/jcm15093328

**Published:** 2026-04-27

**Authors:** Abdulmonem Alsiddiky, Sultana Borai, Sara N. Albqami, Musab Alageel, Abdurahman Addweesh, Nouf Abdulaziz Altwaijri

**Affiliations:** 1 Department of Orthopedics, College of Medicine, King Saud University, Riyadh 12372, Saudi Arabia; 2 Orthopedic Surgery, King Abdullah Specialist Children’s Hospital, Ministry of National Guard Hospital Affairs, Riyadh 14611, Saudi Arabia; 3 Orthopedic Surgery Department, King Saud Medical City, Riyadh 12746, Saudi Arabia

**Keywords:** shoulder level, scoliosis, idiopathic scoliosis

## Abstract

**Background:** Posterior spinal fusion is the mainstay of treatment for Cobb angle over 50 degrees with satisfactory long-term results. In the surgical management of scoliosis, surgeons usually focus on the amount of coronal curvature correction because it can determine the surgical outcome. Nevertheless, there are many factors that contribute to patients’ satisfaction after surgery, and achieving shoulder balance is one of the most vital factors of a successful surgery. Our objective is to study the differences in managing idiopathic scoliosis with pedicle screws versus hybrid fixation with regard to shoulder imbalance postoperatively. **Methods:** Continuous variables were described using mean and standard deviation, whereas categorical variables were described using frequencies. The association between predictor independent variables with the analyzed outcomes were expressed as (beta coefficients) with their associated 95% confidence intervals. The Alpha significance level was considered at 0.050 level. **Results:** The mean angle of the clavicle measured a significant drop post-surgery compared to their pre-surgical measured mean clavicular angle, *p*-value < 0.001, and so did the coracoid height difference: *p*-value < 0.001. Furthermore, the participants had measured a significantly lower mean angle of the clavicle compared to their baseline; *p*-value = 0.029, regardless of their surgery type. The participants mean measured coracoid height difference score had correlated positively with their mean angle of the clavicle: beta coefficient = 1.654, *p*-value < 0.001; when the coracoid height difference increased, so did the mean angle of the clavicle. **Conclusions:** Posterior spinal fusion is effective in correcting coronal curvature and improving radiographic shoulder asymmetry in AIS. Significant improvements were observed in Cobb angle, clavicle angle, and coracoid height difference, with pedicle screw constructs providing superior curve correction. These findings reinforce the value of individualized surgical planning that considers coronal, sagittal, and cosmetic alignment goals.

## 1. Introduction

In general, scoliosis is described as a lateral curve of the spine of over 10 degrees with vertebral rotation [1,2,3,4,5,6]. Naturally, idiopathic scoliosis simply means that the etiology behind it is undetermined and unrelated to any specific congenital, syndromic, or neuromuscular conditions [7,8,9,10]. In addition, there are multiple factors that can lead to progression. Lonstein et al. concluded that the incidence of progression is associated with the age of patients at presentation, pattern and magnitude of the curve, Risser sign and the menarchal status of the patients [11]. According to Tan et al., the most important predictive factor for curve progression to a magnitude over 30° at skeletal maturity was curve magnitude at first presentation [12].

As for the therapeutic options, they include bracing, operative or conservative management [7,8,9,10] with a general goal to maintain the curves under 50 degrees at maturity [13,14,15]. Posterior spinal fusion is the mainstay of treatment for a Cobb angle over 50 degrees with satisfactory long-term results [16,17,18]. In the surgical management of scoliosis, surgeons usually focus on the amount of coronal curvature correction because it can determine the surgical outcome. Nevertheless, there are many factors that contribute to patients’ satisfaction after surgery, for example, back shape improvement, surgical scars, reduced coronal curvature and back pain reduction. Furthermore, the success of surgery is dependent mainly on correctly choosing the fusion levels proximally and distally [19]. Achieving shoulder balance is one of the most vital factors of a successful surgery [20,21,22,23,24,25,26,27,28,29]. The incidence of shoulder imbalance postoperatively in different Lenke subtypes ranged from 20 to 31% in AIS [19,30].

Cosmesis of the back and shoulders are vital to the patients and their parents [21,22,23,26,31,32]. There are multiple domains to study cosmetic disfigurement; the three most vital ones which have been well documented are the radiological measures of trunk and spine alignment such as Cobb angle and apical vertebral translation, patient or parent perception of the appearance of the trunk and body image and objective assessment of trunk symmetry using clinical photography or surface topography [29,33,34]. Some of the risk factors for shoulder imbalance postoperatively have been proposed by various studies and include selection of fusion level, clavicle chest cage angle difference, stiffness of proximal thoracic curve, preoperative T_1_ tilt and clavicle-first rib intersection [25,35,36,37,38,39,40], and despite dramatic improvement in the radiological deformity of the spine, residual cosmetic deformity is not uncommon [26,36,41,42,43,44]. Terheyden et al. noted that the shoulder level preoperatively is the most pertinent factor contributing to the shoulder level postoperatively [19,45] whereas other earlier reports concluded that the postoperative shoulder height correlated with the preoperative SH difference [19,46,47], clavicle height difference [19,20], clavicular angle [19,20,48,49,50,51], CRID [19,20] and first rib angle [19,52].

Other risks of spinal fusion include those of any major surgery, for example, severe blood loss, pancreatitis, urinary infections because of catheterization and obstructive bowel dysfunction because of immobilization during and after surgery [53,54,55,56,57,58,59,60]. Our objective is to study the differences in managing idiopathic scoliosis with pedicle screws versus hybrid fixation with regard to shoulder imbalance and global sagittal and coronal parameters postoperatively.

## 2. Methodology

This is a retrospective observational study that took place in our institute. Our inclusion criteria were adolescence idiopathic scoliosis patients who underwent single posterior spinal correction and fusion with hybrid construct or without, between 2007 and 2019. We excluded patients with scoliosis other than AIS, such as neuromuscular scoliosis and congenital scoliosis. Preoperative and postoperative standing radiographs were used to assess spinal curves using Cobb angle, shoulder imbalance using coracoid height difference (CHD) and clavicular angle (CA), sagittal and coronal balance and thoracic kyphosis.

## 3. Statistical Data Analysis

The mean and standard deviation were used to describe continuous measured variables and the median with the inter-quartile range (IQR) were used to describe the continuous measured variables that showed statistical skewness. The frequencies and percentages were used for describing the categorically measured variables. The Kolmogrove-Smirnove statistical test of normality and the histograms were used to assess the statistical normality assumption for metric variables. The independent samples *t*-test bivariate test was used to assess the statistical significance on mean differences in metric scores between levels of binary dichotomous variables and the chi-squared test of association was used to assess the correlations between categorical variables. The paired samples *t*-test was used to assess the statistical significance of mean differences on metric variables (pre-post tests) and the Chochran’s Q chi-squared test was used to compare the paired samples proportions for significant differences (i.e., to compare significant differences between pre vs. post time points on proportions). The participants’ data was restructured from long to wide data in order to account for the time of assessment as a vector in the analysis, and then the Generalized Multivariable Linear Mixed Regression (GLMixed) Analysis was applied to the participants mean measured scoliosis parameters via regressing these scores against the participants sociodemographic characteristics and the received surgical interventions as well as other relevant factors in order to assess the significant predictors for those scores among scoliosis affected participants. The association between predictor independent variables with the analyzed outcomes was expressed as (beta coefficients) with their associated 95% confidence intervals. The Alpha significance level was considered at 0.050 level. A licensed copy of the SPSS IBM statistical computing program Version 29 was used for the statistical data analysis.

## 4. Results

**Figure 1:**
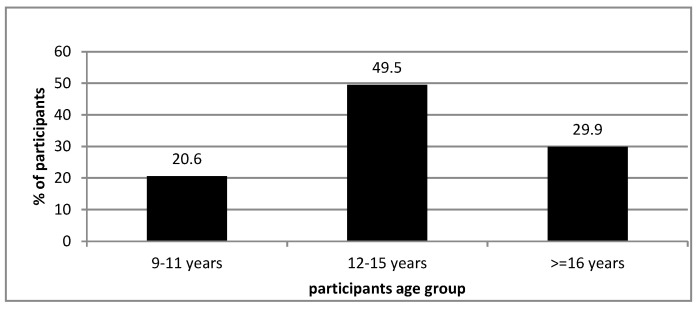
Shows the scoliosis diagnosed participants age groups in our sample.

The medical records of 97 participants with scoliosis requiring surgical interventions were reviewed retrospectively. Table 1 displays the sociodemographic characteristics for those participants, most of them were females 88.7% with a mean age of 14.46 years, SD = 2.69 years (Figure 1). Table 2 displays the descriptive analysis for participants and their required surgical intervention outcomes. A total of 66% required a hybrid scoliosis surgery. Moreover, 12.4% of participants had experienced some surgical complications. The mean surgery duration time in hours for the sample of participants was 5.62 ± 1.49 h and the median Hospital Length of Stay (HLOS) was measured as 6 days with an inter-quartile range of 33 days on average. The mean intraoperative blood loss was 330 milliliters, SD = 241 milliliters, and 6.2% required intravenous blood transfusion intra-operatively to replace blood loss. Only 2.1% required revision surgery.

Table 3 shows the bivariate paired samples *t*-test was used to compare the participants scoliosis-related measurements. The mean angle of the clavicle measured a significant drop post-surgery (mean = 1.54, SD = 1.05) compared to their pre-surgical measured mean clavicular angle (mean = 2.49), *p*-value < 0.001, according to the paired samples *t*-test and regardless of the surgery type. Moreover, the mean coracoid height difference dropped significantly according to the paired samples test post-surgically (mean = 0.69, SD = 0.54) compared to their baseline measured coracoid height difference (Mean = 1.12, SD = 0.91): *p*-value < 0.001. However, according to the Cochran’s Q chi-squared test of repeated measures there had been no significant change in the proportion of participants with anterior to S1 and posterior to S1, and superior anterior, superior mid, and superior posterior located sagittal imbalance, *p*-value > 0.050, respectively. Moreover, according to the Cochran’s Q test the proportion of participants with sagittal imbalance located posterior to S1 had dropped significantly postoperatively (proportion = 36.1%) when compared pre-operative measurements (proportion = 53.6%): *p*-value = 0.010. The paired samples mean thoracic kyphosis angle did not differ significantly before and after scoliosis surgery, *p*-value = 0.141, according to the paired samples *t*-test. However, the participants mean measured lumbar lordosis angle and their mean Cobb angles had declined significantly ppostoperativelycompared to their baseline angles preoperatively, *p*-value < 0.001 according to the paired samples *t*-test.

Table 4 shows bivariate analysis conducted to compare participants undergoing hybrid procedures (*n* = 64) and pedicle screw surgery (*n* = 34) across demographic, clinical, and radiological outcomes. There was no statistically significant association between sex and type of surgical intervention, χ^2^(1) = 2.29, *p* = 0.130, indicating comparable gender distributions across groups. However, participants in the pedicle screw group were significantly older (M = 16.37, SD = 2.73) than those in the hybrid group (M = 13.47, SD = 2.09), t(95) = 5.85, *p* < 0.001. No significant differences were observed between groups in clavicular angle before surgery, t(95) = 1.12, *p* = 0.268, after surgery, t(95) = 0.13, *p* = 0.898, or in clavicular angle change scores, Z = 1.84, *p* = 0.066. Similarly, coracoid height differences—both preoperative, t(95) = 1.06, *p* = 0.291; postoperative, t(95) = 0.81, *p* = 0.426; and change scores, Z = 0.61, *p* = 0.540—did not differ significantly between the two procedures. Sagittal imbalance distributions were also comparable between groups both before, χ^2^(5) = 9.20, *p* = 0.101, and after intervention, χ^2^(5) = 5.87, *p* = 0.319, suggesting no differential effect of surgical approach on sagittal alignment categories. For thoracic kyphosis, no significant differences were found preoperatively, t(95) = 1.12, *p* = 0.224, or postoperatively, t(95) = 1.96, *p* = 0.052, although the latter approached statistical significance. However, the change in thoracic kyphosis angle differed significantly between groups, Z = 2.62, *p* = 0.009, indicating a greater reduction in the hybrid group compared to the pedicle screw group. Lumbar lordosis angles showed no significant differences between groups before surgery, t(95) = 0.32, *p* = 0.753; after surgery, t(95) = 0.70, *p* = 0.483; or in change scores, Z = 0.36, *p* = 0.718. On the other hand, significant differences were observed in Cobb angle measurements. The hybrid group had a higher preoperative Cobb angle (M = 58.29, SD = 15.47) compared to the pedicle screw group (M = 52.26, SD = 10.43), t(88.14) = 2.30, *p* = 0.025, and this difference remained postoperatively, with higher residual angles in the hybrid group (M = 20.55, SD = 18.28) compared to the pedicle screw group (M = 14.24, SD = 9.74), t(94.86) = 2.22, *p* = 0.029. However, the magnitude of Cobb angle correction (change score) did not significantly differ between groups: Z = 0.14, *p* = 0.888. Overall, aside from age, thoracic kyphosis change, and baseline and postoperative Cobb angles, most clinical and radiological outcomes did not significantly differ between the two surgical techniques, suggesting broadly comparable effectiveness across multiple parameters. In order to explain the change in the measured clavicle angle in the pre- and post-operative measurements we utilized the Multivariable Generalized Linear Mixed (GLMixed) Modeling with the Gamma regression of the participants measured mean clavicle angle before and after the surgery against their sociodemographic characteristics, surgical types, complications, outcomes and time of measurement of the angles as a factor in the analysis, as summarized in Table 5. This showed that the participants sex, age and complications did not correlate significantly with their mean angle of the clavicle: *p*-value > 0.050. However, the participants mean Hospital Length of Stay (HLOS) score had correlated positively and significantly with their mean angle of the clavicle: beta coefficient = 0.001, *p*-value = 0.02. Furthermore, the participants had measured significantly lower mean angle of the clavicle compared to their baseline preoperatively on average, beta coefficient = −0.254, *p*-value = 0.029, regardless of their surgery type. The participants mean measured coracoid height difference score had correlated positively with their mean angle of the clavicle: beta coefficient = 1.654, *p*-value < 0.001; when the coracoid height difference increased, so did the mean angle of the clavicle. The participants measured mean lumbar lordosis degrees had correlated slightly negatively with their mean angle of the clavicle: *p*-value = 0.054. An interaction term test was tried between the surgical time and the surgery type and it showed no significant correlation with the participants mean clavicle angle: *p*-value > 0.050. Table 6 displays the GLMixed multivariable analysis for the participants mean measured coracoid height difference score. The analysis findings showed that the participants sex, age, need for revision surgery, intraoperative complications and their operative duration time did not correlate significantly with their mean coracoid height difference: *p*-value > 0.050.

The participants mean clavicle angle had correlated positively, beta coefficient = 0.372, with their mean coracoid height difference: *p*-value < 0.001. Higher clavicle angle predicted significantly higher mean coracoid height difference. Moreover, the analysis model showed that the participants mean lumbar lordosis had converged significantly and positively on their mean thoracic kyphosis angle, beta coefficient = 0.009, *p*-value < 0.001, where greater lumbar lordosis predicted significantly higher mean thoracic kyphosis, as shown in Table 7.

Table 8 displays the multivariable GLMixed modeling results for the participants mean measured lumbar lordosis. The analysis findings showed that female participants had measured significantly higher mean lumbar lordosis degrees scores compared to males on average: beta coefficient = 0.207, *p*-value = 0.001. Interestingly, the participants mean lumbar lordosis was found to be significantly lower postoperatively compared to baseline, beta coefficient = −0.235, *p*-value < 0.001, suggesting a significant drop in the participants mean lumbar lordosis postoperatively.

The participants mean clavicle angle did not correlate significantly with their mean lumbar lordosis, but their mean thoracic kyphosis had converged positively and significantly with their mean lumbar lordosis, beta coefficient = 0.007, *p*-value < 0.001. The children’s experienced intraoperative complications did not correlate significantly with their mean lumbar lordosis.

**Figure 2:**
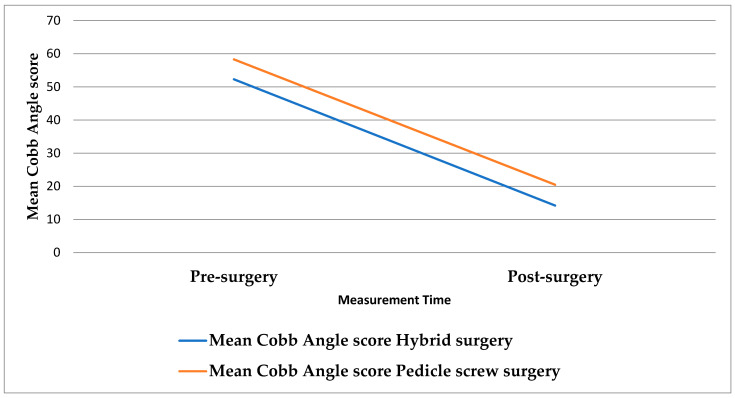
Shows the association between surgery type and children’s mean Cobb angle pre- and post-surgery.

The multivariable GLMixed modeling analysis results of the children’s mean Cobb angle score are displayed in Table 9. The participants mean Cobb angle was found to be significantly lower postoperatively compared to baseline, beta coefficient = −37.837, *p*-value < 0.001. Moreover, the participants who received a Pedicle screw procedure had a significantly lower mean Cobb angle score compared to those who had underwent a Hybrid surgery: beta coefficient = −0.9710, *p*-value = 0.003. The participants who experienced intraoperative complications had measured significantly lower mean Cobb’s angle score, beta coefficient = −6.347, *p*-value = 0.007, compared to those who had not experienced intraoperative complications. The interaction effect test between the surgery types and the surgical time showed no statistically meaningful differences, suggesting that both surgical types resulted in a nearly equal decline in the participants mean Cobb angle postoperatively compared to their baseline. The interaction effect was dismissed from the analysis model to maintain parsimony of the model due to the limited sample size: see Figure 2.

## 5. Discussion

This study evaluated the impact of posterior spinal fusion on shoulder asymmetry in adolescents with idiopathic scoliosis (AIS), using objective radiographic measures including clavicle angle (CA), coracoid height difference (CHD), Cobb angle, thoracic kyphosis, lumbar lordosis, and sagittal balance zones. Our findings support the existing literature showing that surgical correction significantly improves spinal alignment and contributes to better shoulder balance, although individual variables respond differently to surgical intervention.

The mean clavicle angle significantly decreased postoperatively from 2.49° to 1.54° (*p* < 0.001), indicating improved horizontal shoulder symmetry. This aligns with prior studies associating reduced CA with improved cosmetic outcomes and patient satisfaction [19,25,48]. Kuklo et al. and Terheyden et al. have emphasized the clinical relevance of CA in evaluating shoulder level postoperatively [19,45,48]. In our multivariate regression, a strong positive correlation between CA and CHD (β = 1.654, *p* < 0.001) was evident, reinforcing the idea that both parameters reflect complementary aspects of shoulder balance. Interestingly, longer hospital stays were associated with greater postoperative clavicle angles, potentially indicating more complex cases or extended recovery.

Coracoid height difference (CHD) also improved significantly postoperatively, decreasing from 1.12 cm to 0.69 cm (*p* < 0.001), a change that supports the efficacy of posterior fusion in reducing vertical shoulder imbalance. CHDs greater than 1 cm are often clinically visible, and our findings suggest that most patients achieved correction within cosmetically acceptable limits [19,26,30]. The observed correlation between CA and CHD strengthens the validity of using both as dual markers of shoulder-level asymmetry.

The Cobb angle decreased significantly from 56.24° to 18.40° (*p* < 0.001), demonstrating successful coronal curve correction. This magnitude of change is comparable to results reported in multicenter studies utilizing segmental pedicle screw constructs [13,25,50]. Notably, patients who underwent pedicle screw instrumentation achieved greater postoperative correction compared to those treated with hybrid constructs (14.24° vs. 20.55°, *p* = 0.029), reinforcing findings by Suk et al. that segmental screws offer superior biomechanical control [36].

Thoracic kyphosis, however, did not significantly change following surgery (36.63° to 34.33°, *p* = 0.141). This aligns with the work of Lenke and Bridwell, who reported that posterior spinal fusion without kyphosis-specific contouring may maintain but not restore physiological thoracic curvature [15,44]. Importantly, our analysis demonstrated a strong positive correlation between thoracic kyphosis and lumbar lordosis (β = 0.009, *p* < 0.001), confirming their interdependent biomechanical relationship and the importance of preserving sagittal harmony during correction.

Lumbar lordosis decreased significantly postoperatively from 54.19° to 43.41° (*p* < 0.001), suggesting a potential loss of compensatory sagittal curvature following coronal correction. While this trend has been variably reported in the literature, our findings are consistent with those of Ghandhari et al. and Pepke et al., who noted that changes in lumbar lordosis may reflect compensatory adaptation or alignment shifts due to fusion-level and construct rigidity [7,13]. No significant difference in lordosis changes was found between surgical techniques, although females showed higher postoperative values, in agreement with known pelvic morphology differences.

Among sagittal balance categories, only the “posterior to S1” group showed a statistically significant postoperative reduction (from 53.6% to 36.1%, *p* = 0.010), indicating partial improvement in global sagittal alignment. Other sagittal imbalance distributions did not significantly change, which is consistent with previous studies showing that sagittal correction is often a secondary goal in AIS surgery unless specifically planned for [15,44,59]. These findings highlight the need to incorporate sagittal alignment targets more proactively into surgical planning.

Despite the improvements in CA and CHD, a proportion of patients demonstrated residual shoulder asymmetry postoperatively. This is in line with reports by Qiu et al. and Zhang et al., who found that persistent imbalance can occur even in the presence of excellent radiographic correction, due to preoperative asymmetry, proximal thoracic curve stiffness, or suboptimal fusion-level selection [26,30,45]. Our results reinforce the conclusion that achieving cosmetic symmetry requires attention to preoperative shoulder metrics and tailored surgical strategy, particularly with respect to the upper instrumented vertebra.

Interestingly, intraoperative complications were associated with lower final Cobb angles (β = −6.347, *p* = 0.007). While seemingly paradoxical, this may suggest that in more complex cases, greater intraoperative correction efforts were made, leading to both complications and better alignment outcomes. Importantly, complications did not correlate negatively with shoulder symmetry or sagittal profile, suggesting that satisfactory outcomes are still attainable despite operative challenges when appropriately managed.

Overall, this study confirms that posterior spinal fusion improves both radiographic spinal alignment and shoulder balance in AIS. The findings emphasize the utility of clavicle angle and coracoid height difference as objective, reliable, and clinically relevant markers of cosmetic outcome. They also highlight the importance of incorporating sagittal alignment principles into surgical planning to optimize global spine balance and patient satisfaction.

## 6. Conclusions

Posterior spinal fusion is effective in correcting coronal curvature and improving radiographic shoulder asymmetry in adolescents with idiopathic scoliosis. Significant improvements were observed in Cobb angle, clavicle angle, and coracoid height difference, with pedicle screw constructs providing superior curve correction. However, thoracic kyphosis remained largely unchanged, and lumbar lordosis decreased postoperatively, emphasizing the importance of maintaining sagittal balance. Although shoulder asymmetry improved in most patients, residual imbalance persisted in some, likely influenced by preoperative anatomic factors. These findings reinforce the value of individualized surgical planning that considers coronal, sagittal, and cosmetic alignment goals.

## 7. Limitations

This study is limited by its retrospective design and single-institution sample, which may introduce selection bias and limit generalizability. Additionally, radiographic parameters were the sole indicators of shoulder balance, without the inclusion of patient-reported outcomes or surface-based cosmetic assessments. As a result, subjective perception of shoulder symmetry and satisfaction could not be evaluated. Furthermore, the absence of long-term follow-up data limits conclusions regarding the durability of these corrections, as our average follow up was around 3 years. Future prospective studies incorporating patient-centered metrics, 3D surface topography, and standardized long-term outcomes are necessary to further refine surgical strategies and enhance overall care in AIS.

## Figures and Tables

**Table 1:** Descriptive analysis of the children’s sociodemographic characteristics.

	Frequency	Percentage
Sex		
Male	11	11.3
Female	86	88.7
Age (years), mean (SD)		14.46 (2.69)
Age group		
9–11 years	20	20.6
12–15 years	48	49.5
>=16 years	29	29.9

**Table 2:** Descriptive analysis of the children’s scoliosis surgical interventions and its outcomes.

	Frequency	Percentage
Required type of Scoliosis surgical intervention		
Hybrid procedure	64	66
Pedicle screw surgery	33	34
Post surgical complications		
No	85	87.6
Yes	12	12.4
Duration of the surgery (hours), mean (SD)		5.62 (1.49)
Length of Hospital stay (HLOS) days, median (IQR)		6 (33)
Amount of blood loss intra-operatively (mL), mean (S)		330 (241)
Required blood transfusion intra-operatively		
No	91	93.8
Yes	6	6.2
Need for revision surgery		
No	95	97.9
Yes	2	2.1

**Table 3:** Bivariate analysis of the children’s scoliosis measured parameters before and after the surgery.

	Presurgical	Post-Surgical	Test Statistic	*p*-Value
Mean Angle of the clavicle	2.49 (1.83)	1.54 (1.05)	t = 4.84/df = 96	<0.001
Mean Coracoid height difference	1.115 (0.91)	0.69 (0.54)	t = 4.24, df = 96	<0.001
Sagittal imbalance before intervention				
Anterior to S1	7 (7.2)	6 (6.2)	χ^2^(1) < 0.001, df = 1	1.000
Posterior in S1	13 (13.4)	15 (15.5)	χ^2^(1) = 0.042, df = 1	0.839
Posterior to S1	52 (53.6)	35 (36.1)	χ^2^(1) = 6.56, df = 1	0.010
Superior anterior in S1	7 (7.2)	11 (11.3)	χ^2^(1) = 0.563, df = 1	0.453
Superior mid in S1	8 (8.2)	13 (13.4)	χ^2^(1) = 1.07, df = 1	0.302
Superior posterior in S1	10 (10.3)	17 (17.5)	χ^2^(1) = 1.56, df = 1	0.210
Mean Thoracic kyphosis angle	36.63 (15.41)	34.33 (10.88)	t = 1.48, df = 96	0.141
Mean Lumbar lordosis angle	54.19 (15.53)	43.41 (13.10)	t = 7.19, df = 96	<0.001
Mean Cobb angle	56.24 (14.19)	18.40 (16.12)	t = 29.71, df = 96	<0.001

**Table 4:** Bivariate analysis of the participants’ surgical interventions used for the scoliosis repair.

	Scoliosis Type of Sugrical Intervention			
	Hybrid Proedure, *n* = 64	Pedicle Screw Surgery, *n* = 34	Test Statistic	*p*-Value
Sex				
Male	10 (15.6)	1 (3)	Χ^2^(1) = 2.29	0.130
Female	54 (84.4)	32 (97)		
Age (years)	13.47 (2.09)	16.37 (2.73)	t = 5.85, df = 95	<0.001
Mean angle of the clavicle before operative intervention	2.64 (1.76)	2.21 (1.95)	t = 1.12, df = 95	0.268
Mean angle of the clavicle after operative intervention	1.54 (1.13)	1.57 (0.90)	t = 0.128, df = 95	0.898
Clavicular angle change score (post-pre), mean (SD)	−1.10 (1.76)	−0.640 (2.20)	Z = 1.84, df = 97	0.066
Mean coracoid height difference before operative intervention	1.19 (0.96)	0.97 (0.82)	t = 1.06, df = 95	0.291
Mean coracoid height difference after operative intervention	0.730 (0.82)	0.64 (0.45)	t = 0.81, df = 95	0.426
coracoid height difference score, mean (SD)	−0.455 (1.02)	−0.341 (0.85)	Z = 0.613, df = 97	0.540
Sagittal imbalance before intervention				
Anterior to s1	5 (7.8)	2 (6.1)	Χ^2^(5) = 9.20	0.101
Posterior in S1	6 (9.4)	7 (21.2)		
Posterior to S1	33 (51.6)	19 (57.6)		
Superior anterior in S1	7 (10.9)	0		
Superior mid in S1	5 (7.8)	3 (9.1)		
Superior Posterior in S1	8 (12.5)	2 (6.1)		
Sagittal imbalance after intervention				
Anterior to s1	4 (6.3)	2 (6.1)	Χ^2^(5) = 5.87	0.319
Posterior in S1	10 (15.6)	5 (15.2)		
Posterior to S1	26 (40.6)	9 (27.3)		
Superior anterior in S1	9 (14.1)	2 (6.1)		
Superior mid in S1	6 (9.4)	7 (21.2)		
Superior Posterior in S1	9 (14.1)	8 (21.2)		
Mean Thoracic kyphosis angle before intervention	38.01 (15.99)	33.98 (14.1)	t = 1.12, df = 95	0.224
Mean Thoracic kyphosis angle after intervention	32.79 (10.45)	37.31 (11.18)	t = 1.96, df = 95	0.052
Thoracic kyphosis angle change score, mean (SD)	−5.21 (15.3)	3.33 (13.66)	Z = 2.62, df = 97	0.009
Mean Lumbar lordosis angle before intervention	53.011 (16.18)	56.49 (14.18)	t = 0.315, df = 95	0.753
Mean Lumbar lordosis angle after intervention	41.27 (12.10)	47.55 (14.05)	t = 0.704, df = 95	0.483
Lumbar lordosis change score, mean (SD)	−11.74 (16.73)	−8.984 (9.94)	Z = 0.362,df = 97	0.718
Mean Cobb angle before intervention	58.29 (15.47)	52.26 (10.43)	t = 2.30, df = 88.14	0.025
Mean Cobb angle after intervention	20.55 (18.28)	14.24 (9.74)	t = 2.22, df = 94.86	0.029
Cobb angle change score, mean (SD)	−37.75 (13.73)	−38.015 (10.03)	Z = 0.141, df = 97	0.888

**Table 5:** Multivariable Generalized Linear Mixed Regression analysis of the patients mean angle of the clavicle.

	Beta Coefficient	95% C.I. for Beta Coefficient		*p*-Value
		Lower	Upper	
Intercept	1.067	0.455	1.678	0.001
Sex = Female	0.036	−0.269	0.341	0.817
Age (years)	0.126	−0.163	0.415	0.392
Experienced intraoperative complications	0.102	−0.166	0.371	0.453
Hospital Length of Stay days	0.001	0.000	0.002	0.002
Time of measurement = Post-surgical	−0.254	−0.481	−0.026	0.029
Intraoperative Hours	−0.058	−0.136	0.019	0.139
Type of surgery received = Pedicle screw surgery	0.083	−0.161	0.328	0.502
Coracoid height difference degrees	1.654	1.438	1.869	<0.001
Lumbar lordosis degrees	−0.008	−0.016	.000	0.054

Dependent outcome variable = aean angle of the clavicle. Probability distribution: Gamma link function: Identity.

**Table 6:** Multivariable Generalized Linear Mixed Regression analysis of the patients mean coracoid height difference score.

	Beta Coefficient	95% C.I. for Beta Coefficient		*p*-Value
		Lower	Upper	
Intercept	0.030	−0.238	0.299	0.823
Sex = female	0.060	−0.087	0.206	0.421
Age (years)	0.011	−0.153	0.176	0.891
Need for surgical revision = Yes	0.149	−0.133	0.432	0.293
Experienced intraoperative complications = Yes	0.059	−0.068	0.186	0.360
Intraoperative Hours (surgery duration)	0.010	−0.031	0.051	0.637
Time = Post-surgical	−0.012	−0.115	0.092	0.824
Type of surgery received = Pedicle screw surgery	−0.074	−0.208	0.061	0.279
Clavicle angle	0.372	0.330	0.414	<0.001

Target variable = Mean coracoid height difference degrees score. Probability distribution: Normal link function: Identity.

**Table 7:** Multivariable Generalized Linear Mixed Regression analysis of the patients mean thoracic kyphosis angle score.

	Beta Coefficient	95% C.I. for Beta Coefficient		*p*-Value
		Lower	Upper	
Intercept	3.204	2.803	3.604	<0.001
Sex = Female	−0.068	−0.254	0.118	0.473
Age (years)	−0.007	−0.034	0.019	0.588
Time = Post-surgical	0.063	−0.064	0.190	0.330
Type of surgery received = Pedicle screw surgery	0.007	−0.109	0.124	0.903
Hospital Length of Stay days	0.000	0.000	0.001	0.133
Mean Lumbar lordosis degrees score	0.009	0.004	0.013	<0.001

Target variable = Mean thoracic kyphosis angle degrees score. Probability distribution: Gamma link function: Identity.

**Table 8:** Multivariable Generalized Linear Mixed Regression analysis of the patients mean lumbar lordosis degrees score.

	Beta Coefficient	95% C.I. for Beta Coefficient		*p*-Value
		Lower	Upper	
Intercept	3.585	3.245	3.925	<0.001
Sex = Female	0.207	0.081	0.333	0.001
Age (years)	0.006	−0.012	0.023	0.511
Time = Post-surgically	−0.235	−0.313	−0.156	<0.001
Type of surgery received = Pedicle screw surgery	0.093	−0.024	0.210	0.118
Intraoperative Hours (surgery duration)	−0.022	−0.055	0.012	0.200
Mean Clavicle angle score	−0.023	−0.049	0.002	0.073
Mean Thoracic kyphosis score	0.007	0.004	0.010	<0.001
Experienced intraoperative complications	0.077	−0.029	0.183	0.153

Target variable = Mean lumbar lordosis degrees score. Probability distribution: Gamma, link function: Log.

**Table 9:** Multivariable Generalized Linear Mixed Regression analysis of the patients mean Cobb angle degrees score.

	Beta Coefficient	95% C.I. for Beta Coefficient		*p*-Value
		Lower	Upper	
Intercept	53.931	38.834	69.028	0.000
Sex = Female	−2.617	−10.687	5.454	0.523
Age (years)	−0.794	−7.719	6.131	0.821
Time = Post-surgically	−37.837	−41.917	−33.758	<0.001
Type of surgery received = Pedicle screw surgery	−9.710	−16.037	−3.383	0.003
Intraoperative Hours (surgery duration)	1.948	−0.528	4.425	0.122
Experienced intraoperative complications	−6.347	−10.833	−1.860	0.007

Target variable = Mean Cobb angle. Probability distribution: Normal link function: Identity.

## Data Availability

The original contributions presented in this study are included in the article. Further inquiries can be directed to the corresponding author.

## References

[1] Horne J.P., Flannery R., Usman S. (2014). Adolescent Idiopathic Scoliosis: Diagnosis and Management. Am. Fam. Physician.

[2] Lonstein J.E. (1994). Adolescent idiopathic scoliosis. Lancet.

[3] Smith J.R., Sciubba D.M., Samdani A.F. (2008). Scoliosis: A straightforward approach to diagnosis and management. JAAPA.

[4] Reamy B.V., Slakey J. (2001). Adolescent Idiopathic Scoliosis: Review and Current Concepts. Am. Fam. Physician.

[5] Roach J.W. (1999). Adolescent idiopathic scoliosis. Orthop. Clin. N. Am..

[6] Bunnell W.P. (2005). Selective screening for scoliosis. Clinical Orthopaedics and Related Research.

[7] Pepke W., Almansour H., Lafage R., Diebo B.G., Wiedenhöfer B., Schwab F., Lafage V., Akbar M. (2019). Cervical spine alignment following surgery for adolescent idiopathic scoliosis (AIS): A pre-to-post analysis of 81 patients. BMC Surg..

[8] Menger R.P., Sin A.H. (2018). Scoliosis, Adolescent and Idiopathic.

[9] Zhu H., Li B., Jian Y., Sun Z., Yang Z. (2019). Effectiveness analysis of Lenke type 1 adolescent idiopathic scoliosis with different proximal fixation vertebra. Chin. J. Reparative Reconstr. Surg..

[10] Fruergaard S., Ohrt-Nissen S., Dahl B., Kaltoft N., Gehrchen M. (2019). Neural axis abnormalities in patients with adolescent idiopathic scoliosis: Is routine magnetic resonance imaging indicated irrespective of curve severity?. Neurospine.

[11] Lonstein J.E., Carlson J.M. (1984). The Prediction of Curve Progression in Untreated Idiopathic Scoliosis During Growth. J. Bone Jt. Surg..

[12] Tan K.J., Moe M.M., Vaithinathan R., Wong H.K. (2009). Curve progression in idiopathic scoliosis: Follow-up study to skeletal maturity. Spine.

[13] Ghandhari H., Ameri E., Nikouei F., Haji Agha Bozorgi M., Majdi S., Salehpour M. (2018). Long-term outcome of posterior spinal fusion for the correction of adolescent idiopathic scoliosis. Scoliosis Spinal Disord..

[14] Janicki J.A., Alman B. (2007). Scoliosis: Review of diagnosis and treatment. Paediatr. Child Health.

[15] Tari S.H.V., Mahabadi E.A., Ghandehari H., Nikouei F., Javaheri R., Safdari F. (2015). Spinopelvic Sagittal Alignment in Patients with Adolescent Idiopathic Scoliosis. J. Res. Orthop. Sci..

[16] Xi P., Ffarcs Y., Edgar M.A., Frcs M. (1985). Posterior spinal fusion and instrumentation in the treatment of adolescent idiopathic scolio sis Consultant Anaesthetist. Ann. R. Coll. Surg. Engl..

[17] Collis D.K., Ponseti I.V. (1969). Long-Term Follow-Up of Patients with Idiopathic Scoliosis Not Treated Surgically. J. Bone Jt. Surg..

[18] Moskowitz A., Moe J.H., Winter R.B., Binner H. (1980). Long-Term Follow-Up of Scoliosis Fusion. J. Bone Jt. Surg..

[19] Basu S., Suri T. (2020). Shoulder balance in adolescent idiopathic scoliosis: Current concepts and technical challenges. Indian Spine J..

[20] Hong J.Y., Suh S.W., Modi H.N., Yang J.H., Park S.Y. (2013). Analysis of factors that affect shoulder balance after correction surgery in scoliosis: A global analysis of all the curvature types. Eur. Spine J..

[21] Asher M., Lai S.M., Burton D., Manna B. (2003). The reliability and concurrent validity of the Scoliosis Research Society-22 patient questionnaire for idiopathic scoliosis. Spine.

[22] Asher M.A., Lai S.M., Glattes R.C., Burton D.C., Alanay A., Bago J. (2006). Refinement of the SRS-22 health-related quality of life questionnaire function domain. Spine.

[23] Haher T.R., Gorup J.M., Shin T.M., Homel P., Merola A.A., Grogan D.P., Pugh L., Lowe T.G., Murray M. (1999). Results of the scoliosis research society instrument for evaluation of surgical outcome in adolescent idiopathic scoliosis: A multicenter study of 244 patients. Spine.

[24] Haher T.R., Merola A., Zipnick R.I., Gorup J., Mannor D., Orchowski J. (1995). Meta-analysis of surgical outcome in adolescent idiopathic scoliosis: A 35-year english literature review of 11,000 patients. Spine.

[25] Li M., Gu S., Ni J., Fang X., Zhu X., Zhang Z. (2009). Shoulder balance after surgery in patients with Lenke Type 2 scoliosis corrected with the segmental pedicle screw technique: Clinical article. J. Neurosurg. Spine.

[26] Qiu X.S., Ma W.W., Li W.G., Wang B., Yu Y., Zhu Z.Z., Qian B.P., Zhu F., Sun X., Ng B.K. (2009). Discrepancy between radiographic shoulder balance and cosmetic shoulder balance in adolescent idiopathic scoliosis patients with double thoracic curve. Eur. Spine J..

[27] Raso V.J., Lou E., Hill D.L., Mahood J.K., Moreau M.J., Durdle N.G. (1998). Trunk distortion in adolescent idiopathic scoliosis. J. Pediatr. Orthop..

[28] Sanders J.O., Polly D.W., Cats-Baril W., Jones J., Lenke L.G., O’Brien M.F., Richards B.S., Sucato D.J. (2003). AIS Section of the Spinal Deformity Study Group. Analysis of patient and parent assessment of deformity in idiopathic scoliosis using the Walter Reed Visual Assessment Scale. Spine.

[29] Smith P.L., Donaldson S., Hedden D., Alman B., Howard A., Stephens D., Wright J.G. (2006). Parents’ and patients’ perceptions of postoperative appearance in adolescent idiopathic scoliosis. Spine.

[30] Zhang S.F., Zhang L., Feng X.M., Yang H.L. (2018). Incidence and risk factors for postoperative shoulder imbalance in scoliosis: A systematic review and meta-analysis. Eur. Spine J..

[31] Iwahara T., Imai M., Atsuta Y. (1998). Quantification of cosmesis for patients affected by adolescent idiopathic scoliosis. Eur. Spine J..

[32] Theologis T.N., Jefferson R.J., Simpson A.H., Turner-Smith A.R., Fairbank J.C. (1993). Quantifying the cosmetic defect of adolescent idiopathic scoliosis. Spine.

[33] Venugopal Menon K., Pillay H.M., Tahasildar N., Kumar R.J. (2011). Vital capacity evolution in patients treated with the CMCR brace: Statistical analysis of 90 scoliotic patients treated with the CMCR brace. Scoliosis.

[34] Buchanan R., Birch J.G., Morton A.A., Browne R.H. (2003). Do You See What I See? Looking at Scoliosis Surgical Outcomes Through Orthopedists’ Eyes. Spine.

[35] Jian Y.M., Yang S.H., Hu M.H. (2018). Assessment of Change of Shoulder Balance in Patients with Adolescent Idiopathic Scoliosis after Correctional Surgery. Orthop. Surg..

[36] Suk S.I., Kim W.J., Lee C.S., Lee S.M., Kim J.H., Chung E.R., Lee J.H. (2000). Indications of proximal thoracic curve fusion in thoracic adolescent idiopathic scoliosis: Recognition and treatment of double thoracic curve pattern in adolescent idiopathic scoliosis treated with segmental instrumentation. Spine.

[37] Liu Z., Hu Z., Qiu Y., Zhang Z., Zhao Z., Han X., Zhu Z. (2017). Role of Clavicle Chest Cage Angle Difference in Predicting Postoperative Shoulder Balance in Lenke 5C Adolescent Idiopathic Scoliosis Patients after Selective Posterior Fusion. Orthop. Surg..

[38] Yaszay B., Bastrom T.P., Newton P.O. (2013). Should shoulder balance determine proximal fusion levels in patients with lenke 5 curves?. Spine Deform..

[39] Han X., Liu Z., Qiu Y., Sha S., Yan H., Jin M., Zhu Z. (2016). Clavicle Chest Cage Angle Difference: Is It a Radiographic and Clinical Predictor of Postoperative Shoulder Imbalance in Lenke i Adolescent Idiopathic Scoliosis?. Spine.

[40] Luhmann S.J., Sucato D.J., Johnston C.E., Stephens Richards B., Karol L.A. (2016). Radiographic assessment of shoulder position in 619 idiopathic scoliosis patients: Can T1 tilt be used as an intraoperative proxy to determine postoperative shoulder balance?. J. Pediatr. Orthop..

[41] Lee C.K., Denis F., Winter R.B., Lonstein J.E. (1993). Analysis of the upper thoracic curve in surgically treated idiopathic scoliosis-a new concept of the double thoracic curve pattern. Spine.

[42] Winter R.B. (1989). The idiopathic double thoracic curve pattern: Its recognition and surgical management. Spine.

[43] Lenke L.G., Bridwell K.H., O’brien M.F., Baldus C., Blanke K. (1994). Recognition and treatment of the proximal thoracic curve in adolescent idiopathic scoliosis treated with cotrel-dubousset instrumentation. Spine.

[44] King H.A., Moe J.H., Bradford D.S., Winter R.B. (1983). The selection of fusion levels in thoracic idiopathic scoliosis. J. Bone Jt. Surg..

[45] Terheyden J.H., Wetterkamp M., Gosheger G., Bullmann V., Liljenqvist U., Lange T., Bövingloh A.S., Schulte T.L. (2018). Predictors of shoulder level after spinal fusion in adolescent idiopathic scoliosis. Eur. Spine J..

[46] Zang L., Fan N., Hai Y., Lu S.B., Su Q.J., Yang J.C., Guan L., Kang N., Meng X.L., Liu Y.Z. (2016). Evaluation of the predictors of postoperative aggravation of shoulder imbalance in severe and rigid thoracic or thoracolumbar scoliosis. Eur. Spine J..

[47] Yagi M., Takemitsu M., Machida M. (2013). Chest cage angle difference and rotation of main thoracic curve are independent risk factors of postoperative shoulder imbalance in surgically treated patients with adolescent idiopathic scoliosis. Spine.

[48] Kuklo T.R., Lenke L.G., Graham E.J., Won D.S., Sweet F.A., Blanke K.M., Bridwell K.H. (2002). Correlation of radiographic, clinical, and patient assessment of shoulder balance following fusion Versus nonfusion of the proximal thoracic curve in adolescent idiopathic scoliosis. Spine.

[49] Ilharreborde B., Even J., Lefevre Y., Fitoussi F., Presedo A., Souchet P., Penneçot G.F., Mazda K. (2008). How to determine the upper level of instrumentation in Lenke types 1 and 2 adolescent idiopathic scoliosis: A prospective study of 132 patients. J. Pediatr. Orthop..

[50] Lenke L.G., Betz R.R., Haher T.R., Lapp M.A., Merola A.A., Harms J., Shufflebarger H.L. (2001). Multisurgeon assessment of surgical decision-making in adolescent idiopathic scoliosis curve classification, operative approach, and fusion levels. Spine.

[51] Matsumoto M., Watanabe K., Kawakami N., Tsuji T., Uno K., Suzuki T., Ito M., Yanagida H., Minami S., Akazawa T. (2014). Postoperative shoulder imbalance in Lenke Type 1A adolescent idiopathic scoliosis and related factors. BMC Musculoskelet. Disord..

[52] Smyrnis P.N., Sekouris N., Papadopoulos G. (2009). Surgical assessment of the proximal thoracic curve in adolescent idiopathic scoliosis. Eur. Spine J..

[53] Weiss H.R., Goodall D. (2008). Rate of complications in scoliosis surgery—A systematic review of the Pub Med literature. Scoliosis.

[54] Moe J.H., Bradford D.S., Winter R.B., Lonstein J.E. (1978). Scoliosis: And Other Spinal Defomities.

[55] Farley F.A., Caird M.S. (2001). Pancreatitis after posterior spinal fusion for adolescent idiopathic scoliosis. J. Spinal Disord..

[56] Mehta D.I., Festa C., Dabney K., Theroux M., Miller F. (2001). Acute pancreatitis in children with idiopathic vs neuromuscular scoliosis post surgical repair. Gastroenterology.

[57] Roush T.F., Crawford A.H., Berlin R.E., Wolf R.K. (2001). Tension pneumothorax as a complication of videoassisted thorascopic surgery for anterior correction of idiopathic scoliosis in an adolescent female. Spine.

[58] Shapiro G., Green D.W., Fatica N.S., Boachie-Adjei O. (2001). Medical complications scoliosis surgery. Curr. Opin. Pediatr..

[59] Laplaza F.J., Widmann R.F., Fealy S., Moustafellos E., Illueca M., Burke S.W., Boachie-Adjei O. (2002). Pancreatitis after surgery in adolescent idiopathic scoliosis: Incidence and risk factors. J. Pediatr. Orthop..

[60] Sucato D.J., Girgis M. (2002). Bilateral Pneumothoraces, Pneumomediastinum, Pneumoperitoneum, Pneumoretroperitoneum, and Subcutaneous Emphysema Following Intubation with a Double-Lumen Endotracheal Tube for Thoracoscopic Anterior Spinal Release and Fusion in a Patient with Idiopathic Scoliosis. J. Spinal Disord. Tech..

